# Differences in adiposity and diet quality among individuals with inflammatory bowel disease in Eastern Canada

**DOI:** 10.1371/journal.pone.0200580

**Published:** 2018-07-19

**Authors:** Vanessa DeClercq, Morgan G. I. Langille, Johan Van Limbergen

**Affiliations:** 1 Population Cancer Research Program, Dalhousie University, Halifax, Nova Scotia, Canada; 2 Department of Pharmacology, Dalhousie University, Halifax, Nova Scotia, Canada; 3 Department of Pediatrics, Division of Pediatric Gastroenterology & Nutrition, IWK Health Centre, Dalhousie University, Halifax, Nova Scotia, Canada; 4 Department of Medicine, Division of Digestive Care and Endoscopy, Dalhousie University, Halifax, Nova Scotia, Canada; Institute of Subtropical Agriculture, Chinese Academy of Sciences, CHINA

## Abstract

The objective of the current study was to characterize the relationship between diet quality and body composition in participants living with IBD, specifically Crohn’s disease (CD) or ulcerative colitis (UC), in Atlantic Canada. Participants from the Atlantic Partnership for Tomorrow’s Health (PATH) study are residents of one of the four Atlantic Canada provinces. Participants who completed the dietary questionnaire and had body composition measured were included in the study (n = 12,462 without IBD, n = 111 CD, n = 119 UC). A greater number of participants with IBD reported having multiple chronic conditions compared to those without IBD. Those with UC had statistically higher body weight and body mass index (BMI) compared to those without IBD. Overall, significant positive correlations were observed between adiposity and servings of refined grains, and meats and alternatives such as eggs and fish, whereas negative correlations were observed with servings of vegetables, fruit, whole grains, and alternatives such as tofu, and nuts/seeds. Participants with IBD (both CD and UC) consumed more refined grains than those without IBD. Using logistic regression analysis, participants consuming more servings of vegetables and whole grains were less likely to have CD where as those consuming more serving of fruit and bean/legumes were less likely to have UC. In the Atlantic PATH cohort, which includes a region of the world with a high incidence of IBD, distinct differences in adiposity and diet quality were observed in individuals with specific types of IBD compared to those without. There is a need for collaborative efforts to address weight management and diet quality issues in those living with IBD in the Atlantic Canadian region.

## Introduction

Both the incidence and prevalence of inflammatory bowel diseases (IBD), including Crohn’s disease (CD), ulcerative colitis (UC) and IBD-type undetermined IBDU are increasing worldwide. [1] There is great concern about the strain on healthcare systems, as well as the increased risk of colorectal cancer and long-term morbidity of those living with IBD. The number of individuals living with IBD in Canada is among some of the highest in the world [1,2], and in 2012, the estimated costs of IBD in Canada were $2.8 billion annually [3].

Patients with CD often have abnormal mesenteric adipose tissue, known as fat-wrapping or "creeping fat", and mesenteric fat has been found to be a source of pro-inflammatory cytokines [4]. Patients with CD are known to have a higher ratio of intra-abdominal to total abdominal fat, and higher visceral adipose tissue area, than controls. This excess adipose tissue around the intestine contributes to a pro-inflammatory environment, secreting cytokines and adipokines. This excess inflammation is of concern because individuals with IBD are found to have a higher prevalence of inflammatory diseases and chronic comorbidities [5,6].

In addition to excess adipose tissue, diet quality also plays a role in the regulation of the intestinal microbiome and can further augment the associated inflammatory conditions [7–10]. Diet quality deserves increased attention not only for modulating inflammation, but also to avoid nutritional deficiencies and manage symptoms in those with IBD [7,11]. However, there is a lack of large epidemiological studies that have assessed modifiable inflammatory variables such as diet and adiposity in individuals with IBD. Furthermore, exercise has also been found to reduce inflammation [12] and is important for maintaining muscle mass and strength in those with IBD. However, maintaining levels of regular physical activity may be challenging when trying to manage the symptoms of IBD.

Thus, the aim of the current study was to characterize the relationship between body composition and lifestyle behaviours such as physical activity and diet in participants with CD, UC, or without IBD from Atlantic Canada, a region of the country that not only has the highest incidence in the country [13,14] but highest in the world [2]. We hypothesized that participants with IBD will have poorer diet quality and greater abdominal fat mass compared to those without IBD.

## Materials and methods

### Study population

Participants age 30 to 74 years and residents of one of the four Atlantic Canada provinces were eligible to participant in the Atlantic Partnership for Tomorrow’s Health (PATH) study. All participants provided written informed consent prior to data/sample collection. A total of 31,173 participants that were recruited into the Atlantic PATH cohort between 2009–2015 completed the baseline questionnaire which included demographic information, anthropometric data, lifestyle behaviours and medical history (e.g. IBD, n = 614) as previously described [15]. Research Ethics Boards in each Atlantic province (New Brunswick: Horizon Health Network and Vitalite Health Network; Nova Scotia: Nova Scotia Health Authority Research Ethics Board and IWK Research Ethics Board; Newfoundland and Labrador: Health Research Ethics Board Newfoundland; Prince Edward Island: Health Prince Edward Island) approved the original data and sample collection procedures.

A subset of participants completed a dietary questionnaire and had physical measures (height, weight, grip strength, and body composition) taken. To address the aim of the study, the current project involves a sub-sample of Atlantic PATH participants, who had both completed the dietary questionnaire and had body composition measured and had indicated one specific type of IBD. To allow for comparisons between CD and UC, participants that indicated having both CD and UC were excluded (n = 27). Thus, the subsample included a total of 12,802 participants (n = 12,568 without IBD, n = 114 CD, n = 120 UC), showing relative enrichment of IBD within our cohort as compared with reported prevalence data exceeding 0.3% in North America [2,16].

### Assessment of demographic and lifestyle behaviors

Demographic (sex, age, province, urban/rural, education, income) and lifestyle data (sleep, physical activity, smoking, and alcohol consumption) were assessed. The urban/rural classification was based on previously published work in which the Postal Code Conversion File Plus (PCCF+, version 6C, Statistics Canada) was used to classify study participants as living in urban or rural [17]. The level of education completed by participants was categorized as high school or less, college level, and university level or higher and for income as <$10000, $10000–24999, $25000–49999, $50000–74999, $75000–99999; $100000–149999, $150000–199999 and >$200000. Hours of sleep per night were categorized as <5, 5–7, 7–9, 9–11 and > 11 hours. Information on the physical activity levels of participants were collected using open-ended questions in the International Physical Activity Questionnaire (IPAQ) [18]. For each participant, a total physical activity score in metabolic equivalents of a task (MET)-minutes per week was calculated according to the IPAQ scoring protocol and then sex-specific total MET scores were ranked into tertiles and levels of total physical activity were classified as low, medium and high by the MET score tertiles [19,20]. For smoking behaviour, participants were categorized as current smoker, former smoker or never smoked. Participants were classified as an abstainer, occasional drinker (<3 times/month), regular drinker (1–3 times/week), and habitual drinker (4–7 times/week) of alcohol consumption.

### Assessment of adiposity

Anthropometric data, including height, weight, waist and hip circumference were used to calculate BMI and waist-to-hip ratio. Participants with a BMI of <18.5, 18.5–24.9, 25.0–29.9 and ≥30.0 kg/m^2^ were considered underweight, normal weight, overweight and obese, respectively [21]. Body composition was assessed by Bioelectrical impedance using a Tanita Segmental Body Composition Analyzer (Tanita BC-418) and fat mass data are presented as weight in kg.

### Assessment of diet quality

Diet quality data from participants in the cohort has been previously published [22]. In brief, food frequency questionnaires were used to assess dietary habits, with each participant indicating the frequency with which they usually consumed each item, choosing rarely/never, servings per day, servings per week, or servings per month. Participants also indicated the number of servings customarily consumed in a typical day for fruits and vegetables, milk and dairy products, grains, meat and alternatives.

### Statistical analysis

All statistical analyses were performed with IBM SPSS Statistics software (version 23). Chi-square analyses were used to determine significant associations between demographic, behavioural and dietary variables in those with and without IBD. Differences in continuous anthropometric data among those with and without IBD were analyzed using ANOVA, if necessary followed by post hoc two-tailed t tests adjusted with a Bonferroni correction for multiple comparisons. Categorical variables were presented as counts (%) and continuous variables were presented as means ± standard deviation. The relationship between diet variables and adiposity was assessed by Pearson correlation coefficients. To examine the association between dietary variables and IBD, logistic regressions were used to calculate odds ratios (OR) with 95% confidence intervals. Models were unadjusted or adjusted for variables with p<0.05 or factors previously reported to impact IBD (smoking, physical activity, obesity, and comorbidities as defined by 2 or more chronic conditions). Chronic conditions include hypertension, myocardial infarction, stroke, asthma, chronic obstructive pulmonary disease, depression, diabetes, liver cirrhosis, chronic hepatitis, irritable bowel syndrome, eczema, lupus, psoriasis, multiple sclerosis, osteoporosis, and arthritis. For the development of the statistical models for predicting the probability of having IBD, demographic variables, lifestyle behaviors, and anthropometric measures were included in the regression models. The model’s good calibration was determined by a non-significant (P>0.05) Hosmer-Lemeshow’s goodness of fit test. Only variables with a p<0.05 and that showed good model fit were retained in the final regression model. All corrected p-values <0.05 were considered statistically significant.

## Results

### Demographic and lifestyle characteristics

A significant association was observed between IBD and smoking status, alcohol consumption and the number of chronic conditions. Most participants without IBD had never smoked whereas a higher percent of those with CD or UC were former smokers. A smaller percent of participants with IBD were regular/habitual alcohol consumers (33% of CD and 40% of UC) compared to nearly half (48%) of those without IBD. Of those with IBD, approximately ¼ of participants had 4 or more chronic conditions compared to less than 5% of those without IBD. There was no statistically significant association between IBD and sex, province, urban/rural, age, education, income, alcohol consumption, and physical activity level, (Table 1).

**Table 1:** Characteristics of participants with and without IBD.

	Without IBD	CD	UC	
	n (%)			P-value
Sex				0.336
Males	3694 (29.6)	30 (27.0)	42 (35.3)	
Females	8768 (70.4)	81 (73.0)	77 (64.7)	
Province				0.175
Nova Scotia	8873 (71.3)	75 (68.2)	75 (63.0)	
New Brunswick	2299 (18.5)	20 (18.2)	23 (19.3)	
Newfoundland and Labrador	1158 (9.3)	13 (11.8)	19 (16.0)	
Prince Edward Island	121 (1.0)	-	-	
Urban	7994 (64.1)	79 (71.2)	76 (63.9)	0.167
Age				0.781
35–39	965 (7.7)	11 (9.9)	6 (5.0)	
40–49	3024 (24.3)	28 (25.2)	27 (22.7)	
50–59	4677 (37.5)	38 (34.2)	51 (42.9)	
60–69	3796 (30.5)	34 (30.6)	35 (29.4)	
Education				0.096
High School or less	2128 (17.1)	26 (23.6)	26 (21.8)	
College level	4223 (34.0)	37 (33.6)	46 (38.7)	
University level	6072 (48.9)	47 (42.7)	47 (39.5)	
Income				0.129
<$25000	1126 (9.0)	10 (9.0)	14 (11.8)	
$25000–49999	1968 (15.8)	20 (18.0)	24 (20.2)	
$50000–74999	2553 (20.3)	23 (20.7)	23 (19.3)	
$75000–99999	2288 (18.4)	25 (22.5)	22 (18.8)	
$100000–149999	2932 (23.5)	24 (21.6)	25 (21.0)	
>$150000	1620 (13.0)	9 (8.1)	11 (9.2)	
Physical Activity Level				0.146
Low	3971 (32.3)	29 (26.6)	30 (25.6)	
Medium	4095 (33.3)	37 (33.9)	50 (42.7)	
High	4233 (34.4)	43 (39.4)	37 (31.6)	
Smoking status				0.005
Never	6520 (52.3)	59 (44.1)	46 (38.7)	
Former	4932 (39.6)	54 (48.6)	65 (54.6)	
Current	1010 (8.1)	8 (7.2)	8 (6.7)	
Alcohol drinking				0.053
Abstainer	1469 (11.8)	18 (16.2)	15 (12.6)	
Occasional drinker	5021 (40.3)	56 (50.5)	56 (47.1)	
Regular drinking	3842 (30.8)	25 (22.5)	30 (25.2)	
Habitual drinker	2130 (17.1)	12 (10.8)	18 (15.1)	
Chronic Conditions				<0.001
0	4400 (35.3)	0 (0.0)	0 (0.0)	
1	4138 (33.2)	30 (27.0)	30 (25.2)	
2	2324 (18.6)	33 (29.7)	37 (31.1)	
3	1037 (8.3)	16 (14.4)	23 (19.3)	
4+	563 (4.5)	32 (28.8)	29 (24.4)	

Abbreviations: CD, Crohn’s disease; UC, ulcerative colitis.

- Data suppressed due to small cell counts

### Adiposity

Several measures of adiposity were higher in participants with IBD (Supporting Information S1 Table) however, only body weight and BMI were statistically significant in participants with UC compared to those without IBD (Table 2). Hand grip strength measurements were assessed because previous research has shown that grip strength correlates well with overall muscle strength [23,24]. Participants with IBD had similar grip strength than those without IBD (Table 2 and S1 Table). There was no difference in height, hip circumference, and the waist-to-hip ratio among participants with and without IBD (S1 Table).

**Table 2:** Anthropometric data, fat mass, and strength of participants with and without IBD.

	mean (SD)		
	Without IBD	CD	UC
Age	53.7 (8.8)	53.5 (9.1)	54.6 (8.1)
Grip Strength, kg	23.0 (18.6)	22.0 (18.9)	23.1 (19.9)
Height, cm	166.5 (8.8)	165.5 (8.8)	166.7 (9.0)
Weight, kg	75.0 (14.8) _b_	76.2 (15.8) _a,b_	78.9 (14.3) _a_
Waist, cm	92.0 (15.2)	94.3 (17.8)	94.5 (12.7)
Hips, cm	104.6 (12.7)	106.2 (17.3)	106.0 (10.2)
BMI, kg/m^2^	27.0 (4.4) _b_	27.8 (5.5) _a,b_	28.3 (4.5) _a_
Waist-to-hip ratio	0.88 (0.09)	0.89 (0.08)	0.89 (0.07)
Total fat mass, kg	24.6 (9.1)	26.0 (10.4)	26.3 (9.1)
Trunk fat mass, kg	13.1 (5.0)	13.6 (5.3)	14.1 (4.5)
Arm fat mass, kg	2.6 (1.3)	2.8 (1.5)	2.8 (1.3)
Leg fat mass, kg	9.0 (3.5)	9.6 (4.0)	9.4 (3.8)

Abbreviations: BMI, Body mass index; IBD, inflammatory bowel disease; CD, Crohn’s disease; UC, ulcerative colitis

Data presented as means (SD).

Values in the same row with a unlike subscript are significantly different (p<0.0.5).

### Diet quality

Overall, very few participants were meeting Canada’s Guidelines for Healthy Eating [25]. Only 11–13% of participants were meeting the recommended servings of fruits and vegetables, 3% were meeting the recommended servings for grain products, 21–24% were meeting the recommended servings of milk and dairy, and 12–20% were meeting the recommended servings of meat and alternatives (Table 3). There was no significant association between those with and without IBD in meeting guidelines (Table 3 and S2 Table).

**Table 3:** Diet quality of participants with and without IBD.

	Without IBD	CD	UC
Meeting Guidelines^a^	**n (%)**		
Fruits & Vegetable	1632 (13.1)	12 (10.8)	13 (10.9)
Grains	365 (2.9)	-	-
Milk & Dairy	3208 (25.7)	23 (20.7)	28 (23.5)
Meat & Alternatives	1685 (13.5)	13 (11.7)	24 (20.2)
Servings per day	**Mean (SD)**		
Total Fruits & Vegetables	5.3 (2.7)	4.9 (2.4)	5.0 (2.5)
Vegetables	2.5 (1.5)	2.2 (1.3)	2.4 (1.5)
Fruit	2.1 (1.4)	2.0 (1.3)	1.8 (1.2)
Vegetable/Fruit juice	0.7 (1.0)	0.7 (1.0)	0.8 (1.1)
Green Vegetables	0.6 (0.9)	0.5 (0.7)	0.6 (0.9)
Total Grains	2.7 (1.8)_b_	2.6 (2.2)_a,b_	3.1 (2.0)_a_
Whole Grains	1.9 (1.5)_a_	1.5 (1.5)_b_	2.0 (1.6)_a_
Refined Grains	0.8 (1.0)_b_	1.1 (1.7)_a_	1.1 (1.2)_a_
Total Dairy Products	2.1 (1.2)	2.1 (1.4)	2.0 (1.1)
Total Meat & Alternatives	4.4 (2.4)	4.3 (2.4)	4.3 (2.6)
Meat & Poultry	1.6 (1.2)	1.6 (1.0)	1.6 (1.0)
Eggs	1.2 (1.2)	1.3 (1.4)	1.3 (1.5)
Fish	0.5 (0.7)	0.4 (0.7)	0.5 (0.7)
Tofu	0.0 (0.2)	0.1 (0.2)	0.0 (0.2)
Beans & Legumes	0.4 (0.6)	0.4 (0.5)	0.3 (0.5)
Nuts & Seeds	0.7 (0.7)	0.6 (0.7)	0.7 (0.8)
Other Diet Components	**n (%)**		
Most often eaten on bread			
Butter	3883 (31.2)	38 (34.2)	33 (27.5)
Olive oil	301 (2.4)	-	-
Margarine	3957 (31.8)	38 (34.2)	47 (39.5)
Low-fat margarine	2858 (22.9)	20 (18.0)	25 (21.0)
None	1463 (11.7)	13 (11.7)	12 (10.1)
Fat/oil most often used cooking			
None	71 (0.6)	-	-
Margarine	448 (3.6)	-	8 (6.7)
Butter	124 (1.0)	-	-
Other	478 (3.8)	-	-
Olive oil	2790 (22.4)	18 (16.2)	26 (21.8)
Canola	850 (6.8)	5 (4.5)	8 (6.7)
Combination of above	7673 (61.6)	78 (70.3)	72 (60.5)
Type of milk usually consumed			
Homogenized	346 (2.8)	5 (4.5)	5 (4.2)
2% cow’s milk	2166 (17.4)	19 (17.1)	25 (21.0)
1% cow’s milk	3856 (30.9)	28 (25.2)	34 (28.6)
Skim cow’s milk	4261 (34.2)	33 (29.7)	36 (30.3)
Soy	417 (3.3)	5 (4.5)	-
Other	533 (4.3)	9 (8.1)	5 (4.2)
Regular soft drink frequency			
Never	5275 (42.3)	53 (47.7)	51 (42.9)
1–3 times per month	4910 (39.4)	44 (39.6)	43 (36.1)
1–4 times per week	1669 (13.4)	8 (7.2)	18 (15.1)
5–7 times per week	295 (2.4)	-	5 (4.2)
>1 per day	313 (2.5)	5 (4.5)	-
Diet soft drink frequency			
Never	7107 (57.0)	65 (58.6)	69 (58.0)
1–3 times per month	3226 (25.9)	28 (25.2)	27 (22.7)
1–4 times per week	1285 (10.3)	9 (8.1)	18 (15.1)
5–7 times per week	385 (3.1)	-	-
>1 per day	459 (3.7)	6 (5.4)	-
Amount of regular or diet soft drinks			
<10 ounces (<1 can/bottle)	9266 (74.4)	90 (81.1)	92 (77.3)
12–16 ounces (1 can/bottle)	3047 (24.5)	20 (18.0)	24 (20.2)
>16 ounces (>1 can/bottle)	149 (1.2)	-	-
Fast food frequency			
Never	1985 (15.9)	16 (14.4)	13 (10.9)
≤ 1 per month	5593 (44.9)	57 (33.3)	54 (45.4)
2–5 times per month	4447 (35.7)	37 (34.3)	48 (40.3)
2–4 times per week	343(2.8)	-	-
≥5 times per week	94 (0.8)	-	-

^a^Meeting Guidelines: based on Canada’s Guidelines for Healthy Eating by sex and age.

Abbreviations: CD, Crohn’s disease; UC, ulcerative colitis

- Data suppressed due to small cell counts.

Values in the same row with a unlike subscript are significantly different (p<0.0.5).

Fruit and vegetable consumption was lower in those with IBD compared to those without IBD (S1 Table), however, fruit and vegetable consumption was not significantly different between those with CD and UC (Table 3). Overall, most participants reported consuming five servings of fruits and vegetables per day. The intake of grains was significantly different among participants with CD, UC, and without IBD. On average, participants without IBD reported consuming 2 servings of whole grains and 1 serving of refined grains per day whereas participants with CD reported consuming significantly less whole grains than both participants with UC and without IBD. Both CD and UC participants consumed significantly more refined grains than participants without IBD (Table 3). Overall, total milk and dairy, and total meat and alternatives did not differ significantly between those with and without IBD (Table 3 and S2 Table). Participants reported consuming an average of two servings of milk and dairy per day, and skim and 1% cow’s milk were the most commonly reported types of milk consumed. Participants reported consuming an average of 4.3 servings total of meat and alternatives per day per day, and this was similar between participants with CD, UC, and without IBD (Table 3). Both diet and regular soft drink consumption were similar between those with and without IBD, with participants most frequently reporting never consuming or only 1–3 times per month. With respect to oils and fats consumed, participants reported using margarine most often on bread and a combination of fat/oils for cooking. There was no association between the type of oil and fat consumed in participants with or without IBD. Fast food frequency was similar among groups with most participants consumed fast food less than once per month (Table 3 and S2 Table).

### Correlation between adiposity and dietary habits

Measures of adiposity such as BMI, total fat mass and trunk mass correlated with servings of fruits/vegetables, specific grains, and meat/alternatives, however, upon closer examination of individual food items distinct patterns were observed. Servings of refined grains, meat/poultry, and eggs were positively correlated with BMI. The same correlations were observed with total fat mass and trunk mass. In contrast, servings of vegetables, fruit, whole grains, tofu, and nuts/seeds were all inversely correlated with BMI. Total fat mass and trunk mass were also negatively correlated with vegetables, fruit, whole grains, and tofu servings (Table 4).

**Table 4:** Association between BMI and servings of dietary variables.

Variable	*R*	Variable	*R*
Total Fruits & Vegetables	−0.056*^,^^†^^,^ ^#^	Total Milk & Dairy	−0.013
Vegetables	−0.062*^,^^†^^,^^#^	Total Meat & Alternatives	0.046*^,^^†^^,^^#^
Fruit	−0.046*^,^^†^^,^^#^	Meat & Poultry	0.088*^,^^†^^,^^#^
Vegetable/Fruit juice	0.004^†^	Eggs	0.034*^,^^†^^,^^#^
Green Vegetables	0.057*^,^^†^^,^^#^	Fish	0.017*
Total Grains	0.013	Tofu	−0.019 *^,^^†^^,^^#^
Whole Grains	−0.028*^,^^†^^,^^#^	Beans & Legumes	0.010
Refined Grains	0.063*^,^^†^^,^^#^	Nuts & Seeds	−0.049*^,^^†^^,^^#^

Abbreviations: BMI, Body mass index

Results are expressed as Pearson correlation coefficients for associations with BMI.

* Indicates statistically significant association with BMI (*p* < 0.05).

^†^ and ^#^ indicate statistically significant associations found between body fat mass and trunk fat mass, respectively (*p* < 0.05), coefficients not shown.

### Association between dietary habits and IBD

Since differences in adiposity were observed in those with UC (Table 3) and significant correlations were detected between diet quality and adiposity (Table 4), we next wanted to determine if there was an association between diet quality and CD or UC. Servings of vegetables and whole grains were negatively associated with CD, whereas refined grains were positively associated (Table 5). Similarly, refined grains were also associated with UC, but in contrast servings of fruit and beans/legumes were negatively associated with UC. No other dietary variables, being a current smoker or engaging in a high level of physical activity were significantly associated with either CD or UC. A negative association was observed between fruit and vegetables with overall IBD (both CD and UC) and a positive association between servings of refined grains and IBD (S3 Table).

**Table 5:** Associations between health behaviours, dietary factors and CD and UC.

	CD		UC	
Variable	Odds ratio	95% CI	Odds ratio	95% CI
Current Smoker	1.387	0.658–2.925	1.668	0.797–3.488
High Level of Physical Activity	0.903	0.580–1.404	1.416	0.924–2.172
Total Fruits & Vegetables	0.934	0.868–1.004	0.948	0.885–1.017
Vegetables	**0.849**	**0.740–0.974**	0.923	0.814–1.047
Fruit	0.942	0.818–1.086	**0.842**	**0.728–0.974**
Vegetable/Fruit juice	0.964	0.789–1.179	1.090	0.921–1.290
Green Vegetables	0.825	0.643–1.057	1.021	0.836–1.247
Total Grains	0.978	0.879–1.089	**1.120**	**1.033–1.226**
Whole Grains	**0.805**	**0.697–0.930**	1.035	0.918–1.168
Refined Grains	**1.270**	**1.111–1.453**	**1.256**	**1.100–1.436**
Total Milk & Dairy	1.009	0.867–1.174	0.919	0.787–1.074
Total Meat & Alternatives	0.978	0.902–1.059	0.979	0.906–1.058
Meat & Poultry	0.969	0.824–1.141	1.007	0.871–1.166
Eggs	1.074	0.924–1.249	1.030	0.883–1.200
Fish	0.834	0.619–1.124	0.915	0.695–1.204
Tofu	1.311	0.700–2.454	0.752	0.267–2.116
Beans & Legumes	0.916	0.662–1.267	**0.663**	**0.459–0.958**
Nuts & Seeds	0.821	0.622–1.084	0.995	0.781–1.269

Abbreviations: CD, Crohn’s disease; UC, ulcerative colitis

Boldface text indicates significant association (*P*<0.05)

## Discussion

Canada has the highest prevalence of IBD worldwide [1,2], and this study highlights distinct differences in adiposity and diet quality in individuals with IBD from the Atlantic regions of Canada, an area of the country with particularly high incidence [2,13,14]. More specifically, body weight and BMI were significantly greater in those with UC compared to those without. BMI positively correlated with servings of refined grains, meat/poultry, eggs and fish, and inversely correlated with servings of vegetables, fruit, whole grains, tofu, and nuts/seeds. Compared to those without IBD, significant differences in the amount of fruits, vegetables and grains were observed in those IBD. Those that consumed a higher number of servings of fruit/vegetables were less likely to have UC/CD, and those with a higher intake of refined grains were more likely to have IBD (either CD or UC) whereas beans and legumes appeared protective against UC only.

The increasing incidence of IBD in developed and developing countries [1], and increased risk of IBD in those that migrate to high-incidence regions suggests that there is likely a common set of environmental factors that are shared among these individuals [7,26]. Some of the common lifestyle factors associated with IBD are smoking, physical activity and diet [27]. While smoking is an important environmental factor in IBD [28] with differential effects in CD and UC [26], we did not observe a significant association with CD or UC in the currently study (although this may be due to an overall small number of smokers in the cohort). The dramatically increasing incidence in children [14], suggests that other factors must play a role in the pathogenesis of IBD.

Previous research findings are mixed regarding physical activity and its association with the onset or development of IBD [29]. On the other hand, those with existing IBD can tolerate moderate exercise without significant gastrointestinal effects and appear to benefit from regular moderate exercise in terms of symptom management, quality of life, and stress levels [29]. In the current study, we observed a similar level of activity among those with CD and UC compared to without IBD. Recent literature suggests that the increased disease activity has a negative impact on physical activity level [30]. If the degree of disease activity varied among participants, it may have influenced activity levels, however, disease activity was not capture in the current study, thus the analysis of associations between disease activity and physical activity level were not possible. While the majority of patients with IBD experienced positive effects of exercise, it is important to note that some individuals with IBD reported a negative impact of exercise, and the overwhelming majority of patients indicated that IBD had temporarily or permanently stopped them from exercising [31]. This is concerning because of the known health benefits of exercise on preventing chronic diseases such as colon cancer and cardiovascular disease [32,33]. Furthermore, the loss of muscle mass and strength is common in patients with IBD [34] and regular exercise may help maintain muscle mass and lean body mass.

Overall changes in BMI reflect alterations in body weight which includes bone, fat and fat-free (lean) body mass, thus limiting its use in detecting a loss of muscle mass or strength. Furthermore, some studies have shown that in patients with IBD, BMI does not correlate well with fat-free mass [35] and research in children suggests that the maintenance of overall body mass or BMI may be due to gains in fat mass [36]. Thus, direct changes in fat mass and muscle mass/strength should be considered in patients with IBD. In the current study, both BMI and fat mass were higher in participants with IBD compared to those without. In addition, mean grip strength, a measure to detect low lean mass [35], was similar in participants with and without IBD. This may be important for controlling disease activity and progression, as contracting muscles produce and secrete myokines which contribute anti-inflammatory and metabolic effects [29]. The actions of myokines may be of significance for counteracting some of the negative actions of adipokines released from adipose tissue [37,38].

Over the past decade, a clear association has been established between diet-induced obesity and the gut microbiota, and research in this area continues to grow as scientists seek to unravel the underlying mechanisms [39,40]. More recently, a greater understanding of the role of the gut microbiota in inflammatory bowel disease has begun to emerge [41] and environmental factors such as diet have been shown to play an important role in gastrointestinal health and modulation of the gut microbiota [7,8,42,43]. Although the role of diet in the prevention and control of IBD is not well understood at this point, it appears that dietary patterns that control long-term inflammation such as non-Westernized diets and diets high in fruit and vegetable, as wells as those that emphasise a plant-based diet may be useful approaches in the future [7,8]. In the current study, several key components of a Western diet (such as high meat, dairy, and sweetened beverages) were similar between participants with and without IBD, however, we did note significant associations between other components such as vegetables, fruit and refined grain intake. Our findings corroborate recent reports in IBD-cohorts showing an association of fibre intake with disease susceptibility (notably CD) and course [9,44,45].

The relationship between diet and the development or management of IBD is complex and varies depending on the type and activity of the disease [46]. A population-based study from Australia identified a significant association between fast food frequency and the risk of both UC and CD but only in UC patients was a protective effect of daily fruit observed [27]. While it may be reasonable to suggest a diet that is considered anti-inflammatory when trying to prevent or reduce the risk of developing IBD, once established the focus on specific food or nutrients may be more important for induction or remission and prevention of relapse: recent data suggest a critical reappraisal of dietary advice regarding reducing fibre intake in IBD is warranted [9,41,47,48].

Major strengths of the current study include a large sample size, measured anthropometric data for assessment of adiposity, and participants from a population that is known worldwide for its prevalence of IBD. Limitations of the current study include that data are self-reported regarding disease diagnosis, physical activity level, and diet history, and its cross-sectional design. Because baseline data was used for this cross-sectional study, we currently can only identify associations, not causality. However, since participants of the Atlantic PATH cohort are part of a large longitudinal study, it will be possible in the future to examine the role of genetic, environmental, behavioural, and lifestyle factors in the development of disease [15].

In conclusion, this is the first population-based Canadian study in adults to investigate the relationship between diet factors, body composition, other comorbidity and the prevalence of specific types of IBD in an area of the county known to have the highest incidence of the disease worldwide. Adiposity was significantly higher in participants with UC compared to those without IBD in the Atlantic PATH cohort. Findings from this study show that most participants with IBD were consuming a diet similar to those that did not have IBD, with the highest proportion of individuals meeting guidelines in the milk and dairy category. In the regression analysis, a protective effect of servings of fruit and bean/legumes was observed against UC whereas vegetables and whole grains servings were protective of CD. Ongoing and future prospective nutritional intervention studies will offer novel insights in a combined approach to optimizing IBD specific outcomes in the context of improving overall population health [49–52].

## Supporting information

S1 Table Anthropometric data, fat mass, and strength of participants with and without IBD. (PDF)

S2 Table Diet quality of participants with and without IBD. (PDF)

S3 Table Associations between health behaviours, dietary factors and IBD. (PDF)
