# Supplementary material for: Differences in adiposity and diet quality among individuals with inflammatory bowel disease in Eastern Canada
Source: PLoS One. 2018 Jul 19;13(7):e0200580. doi: 10.1371/journal.pone.0200580 (PMC6053167; doi:10.1371/journal.pone.0200580)
Supplement: S1 Table — (PDF) [file pone.0200580.s001.pdf]

S1 Table: Anthropometric data, fat mass, and strength of participants with and without IBD.

	mean (SD)	
	**Without IBD**	**IBD**
Age	53.7 (8.8)	54.0 (8.7)
Grip Strength, kg	23.0 (18.6)	22.6 (19.4)
Height, cm	166.5 (8.8)	165.2 (8.9)
Weight, kg	75.0 (14.8)	77.6 (15.0)*
Waist, cm	92.0 (15.2)	94.3 (15.5)*
Hips, cm	104.6 (12.7)	106.2 (14.1)
BMI, kg/m^2	27.0 (4.4)	28.1 (5.0)*
Waist-to-hip ratio	0.88 (0.09)	0.89 (0.07)
Total fat mass, kg	24.6 (9.1)	26.1 (9.7)*
Trunk fat mass, kg	13.1 (5.0)	13.9 (4.9)*
Arm fat mass, kg	2.6 (1.3)	2.8 (1.4)*
Leg fat mass, kg	9.0 (3.5)	9.5 (3.9)*

Abbreviations: BMI, Body mass index; IBD, inflammatory bowel disease
Data presented as means (SD).
*Indicates significant difference from without IBD (p<0.0.5).
