# Supplementary material for: Differences in adiposity and diet quality among individuals with inflammatory bowel disease in Eastern Canada
Source: PLoS One. 2018 Jul 19;13(7):e0200580. doi: 10.1371/journal.pone.0200580 (PMC6053167; doi:10.1371/journal.pone.0200580)
Supplement: S2 Table — (PDF) [file pone.0200580.s002.pdf]

**S2 Table:** Diet quality of participants with and without IBD.

| | **Without IBD** | **IBD** |
|---|---|---|
| Meeting Guidelines[a] | **n (%)** | |
| Fruits & Vegetable | 1632 (13.1) | 25 (10.9) |
| Grains | 365 (2.9) | 6 (2.6) |
| Milk & Dairy | 3208 (25.7) | 51 (22.2) |
| Meat & Alternatives | 1685 (13.5) | 37 (16.1) |
| | | |
| Servings per day | | |
| Total Fruits & Vegetables | 5.3 (2.7) | 4.9 (2.5)* |
| Vegetables | 2.5 (1.5) | 2.3 (1.4)* |
| Fruit | 2.1 (1.4) | 1.9 (1.3)* |
| Vegetable/Fruit juice | 0.7 (1.0) | 0.7 (1.0) |
| Green Vegetables | 0.6 (0.9) | 0.5 (0.8) |
| Total Grains | 2.7 (1.8) | 2.9 (2.1) |
| Whole Grains | 1.9 (1.5) | 1.7 (1.5) |
| Refined Grains | 0.8 (1.0) | 1.1 (1.4)* |
| Total Dairy Products | 2.1 (1.2) | 2.0 (1.2) |
| Total Meat & Alternatives | 4.4 (2.4) | 4.3 (2.5) |
| Meat & Poultry | 1.6 (1.2) | 1.6 (1.1) |
| Eggs | 1.2 (1.2) | 1.3 (1.4) |
| Fish | 0.5 (0.7) | 0.5 (0.7) |
| Tofu | 0.0 (0.2) | 0.0 (0.2) |
| Beans & Legumes | 0.4 (0.6) | 0.3 (0.5) |
| Nuts & Seeds | 0.7 (0.7) | 0.6 (0.7) |
| | | |
| Other Diet Components | | |
| Most often eaten on bread | | |
| Butter | 3883 (31.2) | 71 (30.9) |
| Olive oil | 301 (2.4) | - |
| Margarine | 3957 (31.8) | 85 (37.0) |
| Low-fat margarine | 2858 (22.9) | 45 (19.6) |
| None | 1463 (11.7) | 25 (10.9) |
| Fat/oil most often used cooking | | |
| None | 71 (0.6) | - |
| Margarine | 448 (3.6) | 11 (4.8) |
| Butter | 124 (1.0) | - |
| Other | 478 (3.8) | 6 (2.6) |
| Olive oil | 2790 (22.4) | 44 (19.1) |
| Canola | 850 (6.8) | 13 (5.7) |
| Combination of above | 7673 (61.6) | 150 (65.2) |
| Type of milk usually consumed | | |
| Homogenized | 346 (2.8) | 10 (4.3) |
| 2% cow’s milk | 2166 (17.4) | 44 (19.1) |
| 1% cow’s milk | 3856 (30.9) | 62 (27.) |
| Skim cow’s milk | 4261 (34.2) | 69 (30.0) |
| Soy | 417 (3.3) | 8 (3.5) |
| Other | 533 (4.3) | 14 (6.1) |
| Regular soft drink frequency | | |
| Never | 5275 (42.3) | 104 (45.2) |

| | | |
|---|---|---|
| 1-3 times per month | 4910 (39.4) | 87 (37.8) |
| 1-4 times per week | 1669 (13.4) | 26 (11.3) |
| 5-7 times per week | 295 (2.4) | 6 (2.6) |
| >1 per day | 313 (2.5) | 7 (2.2) |
| Diet soft drink frequency | | |
| Never | 7107 (57.0) | 134 (58.3) |
| 1-3 times per month | 3226 (25.9) | 55 (23.9) |
| 1-4 times per week | 1285 (10.3) | 27 (11.7) |
| 5-7 times per week | 385 (3.1) | - |
| >1 per day | 459 (3.7) | 10 (4.3) |
| Amount of regular or diet soft drinks | | |
| <10 ounces (<1 can/bottle) | 9266 (74.4) | 182 (79.1) |
| 12-16 ounces (1 can/bottle) | 3047 (24.5) | 44 (19.1) |
| >16 ounces (>1 can/bottle) | 149 (1.2) | - |
| Fast food frequency | | |
| Never | 1985 (15.9) | 29 (12.9) |
| ≤ 1 per month | 5593 (44.9) | 111 (48.3) |
| 2-5 times per month | 4447 (35.7) | 85 (37.0) |
| 2-4 times per week | 343(2.8) | - |
| ≥5 times per week | 94 (0.8) | - |

[a]Meeting Guidelines: based on Canada's Guidelines for Healthy Eating by sex and age.
Abbreviations: IBD, inflammatory bowel disease
- Data suppressed due to small cell counts.
*Indicates significant difference from without IBD (p<0.0.5).
