# Supplementary material for: Differences in adiposity and diet quality among individuals with inflammatory bowel disease in Eastern Canada
Source: PLoS One. 2018 Jul 19;13(7):e0200580. doi: 10.1371/journal.pone.0200580 (PMC6053167; doi:10.1371/journal.pone.0200580)
Supplement: S3 Table — (PDF) [file pone.0200580.s003.pdf]

S3 Table: Associations between health behaviours, dietary factors and IBD.

Variable	Odds ratio	95% CI
Current Smoker	1.517	0.896-2.568
High Level of Physical Activity	1.140	0.839-1.550
Total Fruits & Vegetables	**0.940**	**0.894-0.989**
Vegetables	**0.887**	**0.808-0.973**
Fruit	**0.889**	**0.802-0.985**
Vegetable/Fruit juice	1.030	0.904-1.173
Green Vegetables	0.930	0.795-1.088
Total Grains	1.054	0.982-1.130
Whole Grains	0.925	0.843-1.015
Refined Grains	**1.266**	**1.150-1.393**
Total Milk & Dairy	0.962	0.863-1.073
Total Meat & Alternatives	0.979	0.926-1.035
Meat & Poultry	0.991	0.888-1.105
Eggs	1.053	0.946-1.173
Fish	0.876	0.715-1.073
Tofu	1.054	0.604-1.840
Beans & Legumes	0.784	0.613-1.002
Nuts & Seeds	0.910	0.756-1.094

Boldface text indicates significant association (P<0.05)
